# Programmed cell death in human respiratory syncytial virus infection

**DOI:** 10.3389/fcimb.2025.1719352

**Published:** 2025-11-28

**Authors:** Pengyu Yao, Chang Ma, Chao Liu

**Affiliations:** 1 Department of Traditional Chinese Medicine, Jinan Maternity and Child Care Hospital Affiliated to Shandong First Medical University, Jinan, Shandong, China; 2 M. Kandiah Faculty of Medicine and Health Sciences, Universiti Tunku Abdul Rahman, Kajang, Selangor, Malaysia; 3 School of Public Health, Jilin Medical University, Jilin, Jilin, China; 4 Department of Child Health, Jinan Maternity and Child Care Hospital Affiliated to Shandong First Medical University, Jinan, Shandong, China

**Keywords:** respiratory syncytial virus, programmed cell death, apoptosis, necroptosis, pyroptosis, NETosis, ferroptosis

## Abstract

**Purpose:**

Viral infections elicit different forms of host cell death. Indeed, pathways of programmed cell death (PCD) have emerged as central events in the pathogenesis of various viruses. Regulating PCD is also a critical factor in the pathogenesis of respiratory syncytial virus (RSV) infection. This review systematically summarizes the mechanisms and pathological significance of the main PCD pathways related to RSV infection, and aims to deepen the understanding of RSV regulation of PCD. These findings may provide a new insights and potential therapeutic strategies for the precise prevention and treatment of RSV-related diseases.

**Methodology:**

This review provides a comprehensive overview of the historical development of different forms of PCD. A systematic review was conducted across major academic databases, including Elsevier, PubMed, Springer, Google Scholar, and Web of Science, to collect studies related to RSV and PCD, published between the inception of each database and September 2025. The collected studies were then categorized and organized according to PCD type and affected cell type.

**Results:**

In RSV infection, there are a total of five types of PCD identified, including apoptosis, necroptosis, pyroptosis, NETosis, and ferroptosis. Among these, apoptosis is the most frequently regulated form of cell death during RSV infection. A variety of cell types undergo different forms of PCD during RSV infection, including airway epithelial cells, macrophages, neutrophils, dendritic cells (DCs), lymphocytes and neuronal cells. Notably, PCD is related to airway epithelial cells, which is the most common type of PCD.

**Conclusions:**

PCD serves as a central link in the interaction between RSV infection and the host cell. Different PCD pathways (apoptosis, necroptosis, pyroptosis, NETosis, and ferroptosis) play a dual role in RSV pathogenesis; however, the complex relationship between RSV and PCD remains unclear. Further studies are warranted to explore new forms of PCD in RSV infection, as well as the complex relationship between PCD and RSV structure, the cross-regulatory mechanisms between different PCDs, and the variability of PCD in different cell types. Targeted intervention strategies based on PCD pathways may provide new targets and treatment options for RSV-related diseases.

## 1 Background

The global pandemic of infectious diseases made humanity aware of the devastating threat posed by respiratory viruses. While long standing viral diseases (with the exception of smallpox) remain endemic, a new viral diseases have emerged intermittently. These diseases not only inflict significant harm on individuals but also impose substantial burdens on families, healthcare systems, and societies, resulting in considerable economic losses (He et al., 2025). Respiratory syncytial virus (RSV), one of the most common respiratory viral pathogens worldwide, was discovered in 1956 by Dr. Robert Chanock after its isolation from children with upper respiratory tract infection (URTI) (Casadevall et al., 2025). RSV causes acute respiratory tract illness (ARTI) in individuals of all age groups and is responsible for seasonal outbreaks of respiratory illness worldwide (Kuang et al., 2024).

The outcome of a viral infection in the host is determined by diverse cellular responses, including abortive, productive, latent, and destructive infections (Rex et al., 2022). These cellular responses generated, whether the infection remains limited, establishes persistence, or leads to an extensive cytopathic damage. The viral-host interaction should be further elucidated to facilitate the development of novel virus control methods (Yuan et al., 2023). Virus-induced cell death has been thought of as a double edged sword in the inhibition or exacerbation of viral infections (Wang et al., 2022). Cell death is a fundamental physiological process in all organisms, which includes accidental cell death (ACD) and programmed cell death (PCD). In their seminal paper of 1972, Kerr, Wyllie and Currie first collated and defined the distinct morphological features of PCD in different contexts (Kerr et al., 1972). PCD plays a central role in regulating animal development and tissue homeostasis and is also implicated in a broad range of human diseases (Rex et al., 2022). In the setting of viral infection, the primary purpose of PCD is thought to be the restriction of replication and dissemination by depriving viruses of the resources needed to propagate infection. However, previous researchers has revealed that PCD signaling shapes the host response to infection in complex ways (Angel and Daniels, 2022). Multiple viruses, including RSV, have been shown to manipulate various forms of PCD, thereby promoting their replication and persistent infection. Therefore, elucidating on how virus regulates these PCD pathways is crucial to understand its pathogenic mechanisms and may reveal novel therapeutic targets.

The core of RSV pathogenicity lies in the dynamic interplay between viral infection and host defense: on the one hand, the host eliminates infected cells via PCD to restrict viral spread; on the other hand, RSV selectively activates/inhibits specific PCD pathways, thereby triggering excessive inflammation and tissue damage, and severe consequences such as bronchiolitis and asthma sensitization. A growing number of studies have shown that PCD is closely related to RSV infection. In this review, we systematically review the distinct modalities of cell death triggered by RSV infection, clarifying their molecular underpinnings to illuminate viral pathogenesis and identify actionable therapeutic targets.

## 2 Principle of RSV

**Figure 1:**
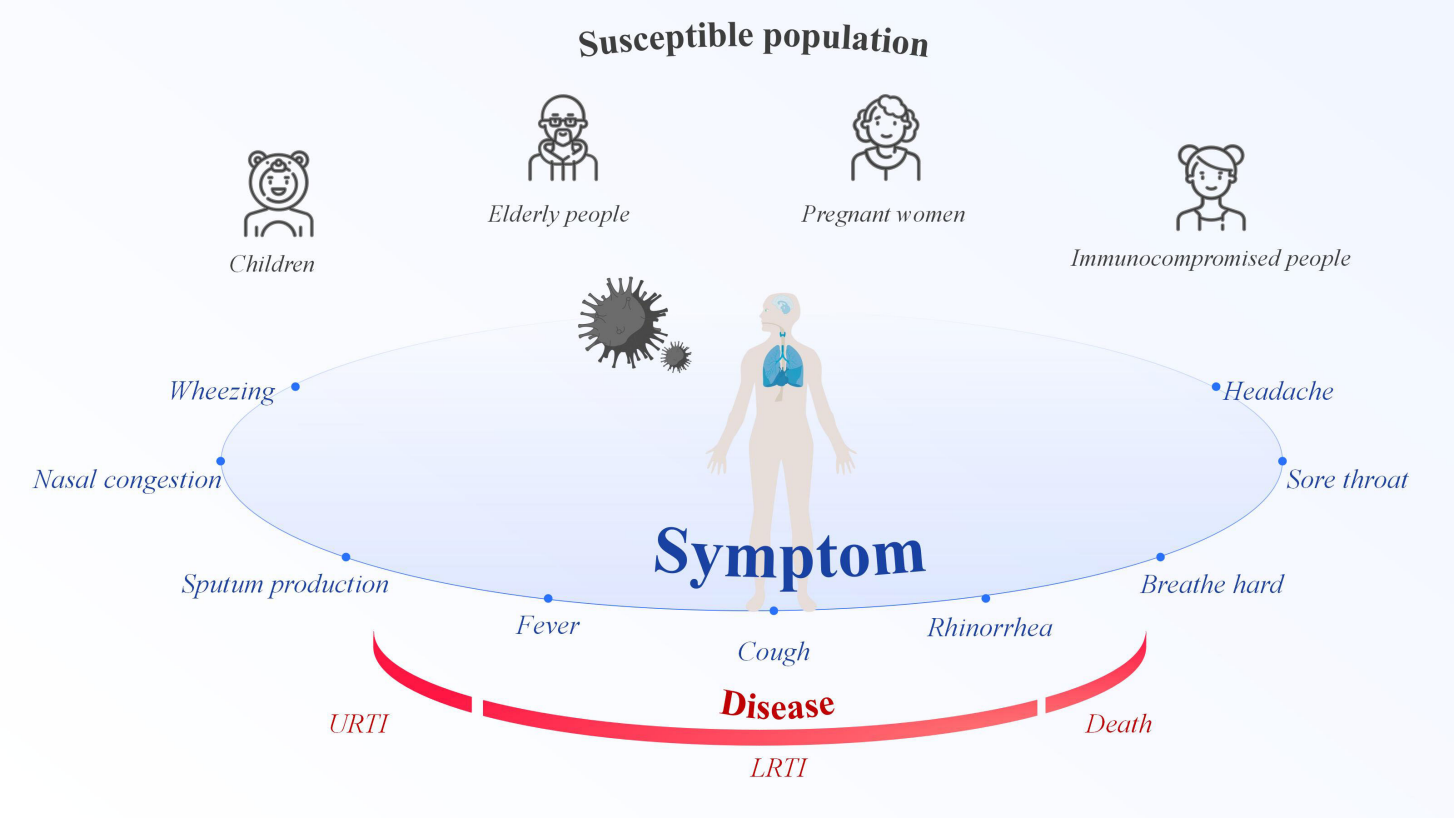
Respiratory syncytial virus symptoms, susceptible populations, and illnesses.

RSV is an enveloped, negative-sense, single-stranded RNA virus belonging to the *Orthopneumovirus genus of the Pneumoviridae family, order Mononegavirales.* RSV first replicates in the nasal turbinates and then propagates to the bronchioles, where increased mucus production, impaired ciliary action, and sloughed epithelial cells obstruct in the lumen. This obstruction results in trapped air, causes alveolar collapse after absorption (Brüssow, 2025). As an RNA virus transmitted via droplets, RSV can infect individuals of all ages, placing everyone at risk of infection (Wildenbeest et al., 2024). However, children, the elderly, pregnant women, and people with weakened immune systems are at the highest risk of RSV infection (**Figure 1**). In 2019, there were 33 million RSV-associated acute lower respiratory infection episodes, 3.6 million hospital admissions, 26,300 in-hospital deaths, and 101,400 overall deaths in children aged 0–60 months, which encompasses the period from birth until the fifth birthday (Li et al., 2022). Infants who are premature, immunocompromised, or who have congenital heart disease, congenital lung disease, or congenital airway defects are most vulnerable to severe RSV-related conditions (Dominoni et al., 2024). RSV is the second leading cause of hospitalization from respiratory infections in the elderly (Barahimi et al., 2023). RSV constitutes a significant cause of respiratory illness and mortality among older adults, that is expanding with considerable impact on healthcare systems worldwide (Duan et al., 2024). For example, In the United States (US), RSV is responsible for an estimated 6,000~10,000 annual deaths among older adults (Kim and Choi, 2024). In the past, little was known about the impact of RSV infection during pregnancy, but this issue has gradually received attention in recent years. Pregnant individuals undergo substantial physiologic and immunologic changes over the course of their pregnancy, increasing their risk for respiratory virus infections (Rumfelt et al., 2025). RSV infection during pregnancy can be clinically severe and may be accompanied by pregnancy complications (Hause et al., 2021). The estimated incidence of prenatal RSV infection is reported to be 2.1 cases per 1,000 person-months, or 26.0 cases per 1,000 person-years (Kenmoe et al., 2023), indicating a considerable disease burden. In addition, RSV infection is significantly associated with immunocompromised populations (such as high-risk allogeneic hematopoietic stem cell transplant and solid organ transplant recipients), resulting in higher morbidity and mortality in this group (Koval and Gonzalez, 2024). Furthermore, natural immunity to RSV is incomplete, and reinfection occurs throughout life (Mazur et al., 2023). As a seasonal disease, RSV infection peaks when temperatures decrease in temperate zones and humidity increases in tropical regions (Chi and Chung, 2023). In temperate regions, RSV epidemics typically last for 2~5 months, with the peak commonly occurring during winter. In the tropics, RSV seasonality varies and have longer durations when compare to temperate regions, however the peak typically aligns with the rainy season (Guo et al., 2024). However, these patterns are not static; in fact, the seasonal prevalence of RSV is changing. For example, in the US, the seasonal circulation of RSV was disrupted during the coronavirus disease 2019 (COVID-19) pandemic. Non-pharmaceutical interventions reduced respiratory virus transmission, leading to an accumulation of susceptible individuals and large epidemics with a typical seasonality (Hamid et al., 2023). The seasonality of respiratory virus epidemics is driven by a complex interplay of environmental and host factors, such as temperature, humidity, sunlight, vitamin levels, and behavior (Moriyama et al., 2020). Human behavior is the key to RSV infection; avoiding crowded places is the best option, and this also applies to most respiratory viruses. Most RSV infections are mild and self-limiting, typically present as mild respiratory illness. Clinical cases shown insufficient to differentiate RSV from other acute respiratory infections (Wildenbeest et al., 2024). The clinical presentation of RSV range from a mild cold to a serious respiratory illness with complications compare to those caused by influenza and other respiratory viruses (Nguyen-Van-Tam et al., 2022). RSV symptoms include rhinorrhea, sore throat, nasal congestion, wheezing, headache, fever, cough, breath hard, and sputum production. The most commonly reported RSV signs and symptoms in high-risk and immunocompromised adult patients were primarily lower respiratory tract infection (LRTI) manifestations (Colosia et al., 2023). For a certain number of people who are at risk of more severe respiratory disease, RSV infection might cause pneumonia or even death.

## 3 The 60-years of programmed cell death

**Figure 2:**
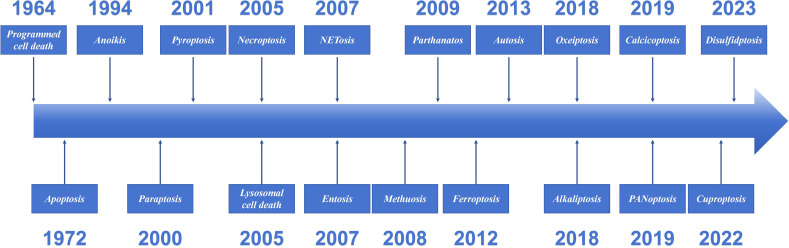
The discovery timeline of programmed cell death.

Cell death is a fundamental physiological process in all living organisms. Since 1960s and 1970s, systematic studies of cell death have begun, making it one of the most rapidly developing areas in biomedicine (Kopeina and Zhivotovsky, 2022). PCD has existed since the earliest stages of cellular evolution. An adult is estimated to lose approximately 10^11^ cells per day through PCD (Newton et al., 2024). However, the phenomenon was not formally summarized until 1964, when R. Lockshin and C. Williams coined the term “Programmed cell death” (Lockshin and Williams, 1964). Over 60 years after its discovery, PCD has emerged as a cornerstone of biomedical research. The significance of apoptosis in the life sciences has been established. In fact, its role and potential value have not yet been fully elucidated. In 2002, The Nobel Prize in Physiology or Medicine was awarded jointly to Sydney Brenner, H. Robert Horvitz and John E. Sulston “for their discoveries concerning genetic regulation of organ development and programmed cell death”. PCD is a cell-initiated, gene-regulated death process, and the discovery and definition of its different forms have gradually become clearer with the progress of research. This review introduced the definitions and characteristics of the main types of PCD identified to date (**Figure 2**).

### 3.1 Apoptosis

The discovery of apoptosis was a fundamental hallmark in the study of cell death and expanded our understanding of various types of cell death (Park et al., 2023). In 1972, *John F. Kerr, Andrew H. Wyllie* and *A. R. Currie* coined the term “apoptosis” to differentiate naturally occurring developmental cell death from the necrosis that results from acute tissue injury (Kerr et al., 1972). Caspase family proteases have long been regarded as the “executioners” of cell death, initiating an irreversible apoptotic cascade upon activation, akin to a domino effect (Li et al., 2025). Apoptosis is carried out by intracellular caspase-3 and caspase-7, which cleave diverse intracellular substrates, leading to cell shrinkage, chromatin fragmentation, membrane blebbing, and breakdown into membrane-wrapped vesicles called apoptotic bodies, which is also a morphological characteristic of apoptotic cells (Ai et al., 2024). Apoptosis is a complex interplay of two main signaling pathways: the intrinsic (mitochondrial) and the extrinsic (death receptor). The extrinsic signaling through death receptors that leads to the formation of the death-inducing signaling complex (DISC), and intrinsic signaling mainly through mitochondria leads to the formation of the apoptosome. Formation of the DISC or apoptosome, respectively, activates initiator and common effector caspases that execute the apoptosis process. The key genes and pathways involved in apoptosis have been increasingly clarified after more than 50 years. The value of apoptosis in explaining the pathological mechanisms of various diseases has been further explored, supporting the development of new therapeutic strategies. Indeed, apoptosis has revolutionized our understanding of cell death and its importance in various physiological and pathological conditions (Capela E Silva and Rodrigues, 2023).

### 3.2 Anoikis

Anoikis was coined by *Frisch* and *Francis* in 1994 (Frisch and Francis, 1994). The term was borrowed from the Greek words meaning “the state of being without a home”. Anoikis, also known as matrix detachment-induced apoptosis or detachment-induced cell death, is directly induced by a disruption in cell-cell attachments or cell-extracellular matrix (ECM) attachments (Wang et al., 2024). The disruption of cytosolic connectors leads to intracellular cytoskeletal and signaling pathway alterations, ultimately activating caspases and initiating PCD, which is an outside-in process that mediated by a caspase cascade reaction (Dai et al., 2023). Anoikis is a distinct form of apoptosis that cannot be simply categorized as traditional apoptosis due to its unique initiation process (Mei et al., 2024). Anoikis maintains tissue homeostasis by eliminating cells that are detached or misplaced due to physiological or pathological conditions.

### 3.3 Paraptosis

Paraptosis is a distinct form of PCD characterized by massive cytoplasmic vacuolation involving the dilation of endoplasmic reticulum (ER) and/or mitochondria. Paraptosis is caspase-independent and lacks the typical morphological changes of apoptosis (Li et al., 2022). Membrane blebbing, chromatin condensation, and nuclear fragmentation—characteristic morphological hallmarks of apoptosis—are absent in the process of paraptosis. It is regulated by several signaling pathways, for instance, those associated with ER stress, calcium overload, oxidative stress, and specific cascades. Paraptosis is a distinct form of non-apoptotic cell death gaining attention for its potential role in cancer therapy and resistance (Kunst et al., 2024). As a novel type of PCD, paraptosis was first identified by *Sperandio* in 2000 (Sperandio et al., 2000). Despite its extensive and long research history in pathophysiology, the incidence of Paraptosis is likely underestimated due to insufficient understanding of its biochemical mechanisms and a lack of specific biomarkers.

### 3.4 Pyroptosis

Pyroptosis is a lytic and inflammatory type of PCD that is triggered by inflammasomes and executed by gasdermin proteins. Pyroptosis is characterized by the activation of inflammatory caspases (mainly caspase-1, 4, 5, 11) and cleavage of various members of the Gasdermin family to form membrane perforation components (Wei et al., 2022). Cell swelling, membrane perforation, and release of cellular contents are its major cell morphological changes. In fact, pyroptosis in infected macrophages was discovered in early 1992 but mistakenly classified as apoptosis (Liu et al., 2024). However, the term “pyroptosis” was first proposed by *Cookson and Brennan* in 2001 to describe this PCD type. Pyroptosis from the Greek roots *pyro*, relating to fire or fever, and *ptosis* (to-sis) to denote a falling, to describe pro-inflammatory programmed cell death (Cookson and Brennan, 2001). In terms of physiology, pyroptosis plays a critical role in host defense against pathogen infection (Rao et al., 2022). As the sole form of PCD that intimately couples cell death with an intense inflammatory response, pyroptosis possesses unique value.

### 3.5 Lysosomal cell death

Lysosomal cell death (LCD) is a form of PCD triggered by increased lysosomal membrane permeabilization (LMP), leading to the cytoplasmic release of lysosomal enzymes and subsequent execution of the cell death cascade. In 1955, *Christian de Duve* discovered lysosomes—a breakthrough that inaugurated a new era in cellular physiology and pathophysiology. He was awarded the *Nobel Prize in Physiology or Medicine* in 1974 for his discovery and characterization of lysosomes as cellular ‘recycling bins’. The concept of LCD was first proposed by *Christian de Duve* (Aits and Jäättelä, 2013). There is much controversy exist when the term “lysosomal cell death” was first proposed. However, it was actually first proposed in 2005, by Kroemer et al., who discussed it as an independent form of PCD (Kroemer and Jäättelä, 2005).

### 3.6 Necroptosis

Necroptosis, as an independent term, was first proposed by *Degterev* et al. in 2005 (Degterev et al., 2005). Necroptosis is a form of programmed inflammatory cell death, characterized by distinct morphological and biochemical hallmarks, including cell membrane rupture, organelle swelling, cytoplasmic and nuclear disintegration, cellular contents leakage, and release of damage-associated molecular patterns (DAMPs), accompanied by the inflammatory responses (Zhu and Wu, 2024). Necroptosis differs from apoptosis and other forms of PCD because it does not rely on caspase activity. Necroptosis is mediated by the necrosome, which consists of mixed lineage kinase domain-like protein (MLKL), receptor-interacting protein kinase 1 (RIPK1), and RIPK3 (Ye et al., 2023).

### 3.7 Entosis

Entosis is one of the known types of vacuole-dependent, non-apoptotic PCD (Gaptulbarova et al., 2024). In 2007, entosis was proposed by *Overholtzer M* (Overholtzer et al., 2007), which was derived from the Greek word that means “inside” or “within”. Entosis is a form of PCD in which one cell inserts itself into a neighboring cell, leading to the eventual death of the invading cell. This creates a characteristic cell-in-cell (CIC) pattern, a phenomenon observed as early as 1891 by *Steinhaus* in tumor samples (Kianfar et al., 2022). During entosis, the inner cell actively invades the host cell, a process driven by the activation of Rho proteins, the formation of adhesion bonds, and actomyosin filaments. Once the phenomenon happened, the inner cell is enclosed within a double-membraned entotic vacuole, featuring a large space between the membranes (Mlynarczuk-Bialy et al., 2020). The four possible fates in entosis are: death of the inner cell, death of the outer cell, death of both cells, or survival of both cells.

### 3.8 NETosis

“NETosis” is a term first introduced by *Steinberg* and *Grinstein* in 2007 to describe the process of programmed neutrophil death associated with the formation of neutrophil extracellular traps (NETs) (Steinberg and Grinstein, 2007). NETosis is characterized by neutrophil cell death accompanied by the release of NETs, auto-inflammatory factors, and antigens. NETs are extracellular web-like structures composed of decondensed DNA decorated with cytosolic and granule proteins. Since it was initially shown that NET formation is accompanied by cell death, this process was termed NETosis (Vorobjeva and Chernyak, 2020). As the most typical and predominant form of cell death in neutrophils, NETosis is the only type of PCD that fights pathogens by releasing NETs.

### 3.9 Methuosis

Methuosis is one of the most recent additions to the list of nonapoptotic cell death phenotypes. The most prominent attribute in cells undergoing this form of death is the accumulation of large fluid-filled cytoplasmic vacuoles that originate from macropinosomes (Maltese and Overmeyer, 2014). Methuosis is a process that lacks the classic morphological features of apoptosis. In 2008, *Overmeyer* et al. discovered that treatment of human glioblastoma cells with a small molecule called MIPP ([3-(2-methyl-1H-indol-3-yl)-1H-pyrrolo[3,2-b]pyridine]) induced a unique type of cell death, coined the term “methuosis” (from the Greek word *methuo*, which means to drink to intoxication) (Overmeyer et al., 2008). Methuosis, as a novel, protease-independent form of cell death that expands the known diversity of cell death networks.

### 3.10 Parthanatos

Parthanatos, also known as PARP-1 dependent cell death, named in 2009 by *Ted Dawson* and *Valina Dawson*, who defined the term “Parthanatos” after *Thanatos*, the personification of death in Greek mythology (David, 2009). Its core mechanism involves hyperactivation of poly (ADP-ribose) polymerase-1 (PARP1). The excessive consumption of NAD+ and ATP subsequently leads to cellular energy crisis. Concurrently, the accumulation of poly (ADP-ribose) (PAR) polymers facilitates the nuclear translocation of apoptosis-inducing factor (AIF), culminating in massive DNA fragmentation and cell demise (Fang et al., 2025). Therefore, the main characteristics of Parthanatos are considered to be PARP1 hyperactivation, PAR polymer accumulation, mitochondrial depolarization, and AIF nuclear translocation, distinct from other cell death pathways, Parthanatos is caspase-independent and does not require Bax or apoptotic activating factor-1 (Apaf-1) (Yang et al., 2024).

### 3.11 Ferroptosis

Ferroptosis is caused by the accumulation of iron and lipid peroxidation, it was first observed in cells treated with erastin, a small-molecule inducer discovered by *Dolma* et al. in 2003 (Dolma et al., 2003). Ferroptotic cells lack of classic apoptosis features like chromatin condensation, apoptotic bodies, autophagosome formation, or nuclear structural changes. However, mitochondria exhibit unique characteristics, including reduced volume, loss of structural integrity, increased membrane density, outer membrane rupture, and decreased or absent cristae (Shen et al., 2025). The concept of this distinct form of regulated, iron-dependent cell death driven by lipid peroxidation, along with the term “Ferroptosis” was later introduced by Dixon et al. in 2012 (Dixon et al., 2012).

### 3.12 Autosis

In 2013, *Liu* and *Levine* identified a novel form of autophagy-induced cell death, termed “autosis”. They found that the selective overactivation of autophagy results in cell death with unique morphological features distinct from those of apoptosis or necrosis (Liu et al., 2013). Before this concept was proposed, the relationship between autophagy and PCD was often considered to be contradictory. Autotic cells are characterized by a dramatic initial increase in cytoplasmic autophagic and empty vesicles, followed by later-stage changes including electron-dense mitochondria, ballooning of the perinuclear space due to the separation of nuclear membranes, and concavity of the nucleus (Schwartz, 2021). Autosis is uniquely characterized by its direct dependence on the overactivation and execution of the autophagic process.

### 3.13 Alkaliptosis

Acid-base homeostasis is essential for normal physiology, cellular metabolism, and function. Alkaliptosis is defined by the lethal rise in intracellular pH, regulated by the interplay of ion channels and transporters in intracellular and extracellular pathways. Compared to other forms of RCD, the induction of alkaliptosis does not involve the commonly recognized cell death effectors, such as caspases and MLKL (Chen et al., 2023). As a type of pH-dependent non-apoptotic PCD, alkaliptosis was first identified by *Tang* et al. in 2018 in pancreatic ductal adenocarcinoma (PDAC) cells (Song et al., 2018).

### 3.14 Oxeiptosis

Oxeiptosis is a novel caspase-independent PCD pathway with apoptotic-like features, first proposed as an academic concept by *Holze* et al. in 2018 (Holze et al., 2018). Reactive oxygen species (ROS) play essential roles in all forms of cell death. ROS-induced cell death includes apoptosis, necroptosis, ferroptosis and autosis (Villalpando-Rodriguez and Gibson, 2021). However, Oxeiptosis operates in a non- inflammatorily, independently of caspases, and is induced by ROS or ROS-generating. Distinguished from other cell death pathways, oxeiptosis shown a unique damage-causing factors pivotal genes, and Kelch-like ECH-associated protein-1 (KEAP1)/Phosphoglycerate mutase family member 5 (PGAM5)/apoptosis-inducing factor mitochondrial associated 1(AIFM1) signaling pathways (Chen et al., 2024). Pathologically high ROS cause oxidative stress, which damages macromolecules (proteins, lipids, DNA) and cellular organelles. This ultimately compromises cellular integrity and leads to apoptosis-like cell death.

### 3.15 PANoptosis

PANoptosis is a unique inflammatory cell death, representing the convergence of apoptosis, pyroptosis, and necroptosis, and plays a crucial role in regulating cell death and immune responses (Gao et al., 2024). Pyroptosis, apoptosis, and necroptosis, three previously considered separate and independent PCD pathways, actually exhibit mechanistic overlap and extensive, multifaceted crosstalk. Crosstalk among pyroptosis, apoptosis, and necroptosis was systematically reported as early as 2016 (Kuriakose et al., 2016). However, the concept of “PANoptosis” was not proposed until 2019 by *R K Subbarao Malireddi* et al (Malireddi et al., 2019). The emergence of the concept of PANoptosis has provided a novel perspective for cell death research, drawing increasing attention to the crosstalk mechanisms underlying PCD.

### 3.16 Calcicoptosis

Disordered cytosolic calcium [Ca^2+^]_(c)_ signaling plays a critical role in cell death (Bhosale et al., 2015). The concept of calcicoptosis as a new type of cell death was recently proposed by Zhang et al. in 2019 (Zhang et al., 2019). The calcicoptosis is a novel form of cell death characterized by calcium-induced damage and oxidative stress, triggered by Ca^2+^ over-loading that leads to mitochondrial dysfunction with excess ROS generation. Under oxidative stress, abnormal function of cellular calcium channels impairs Ca^2+^ concentration modulation, which subsequently causes cell membrane mineralization by Ca_3_(PO_4_)_2_ precipitation (Guo et al., 2022). Calcicoptosis is proposed as a distinct form of PCD, different from other cell types, suggesting cell death induced by calcium.

### 3.17 Cuproptosis

Cuproptosis is a recently discovered form of mitochondria-related cell death triggered by excess Cu^2+^. In 2022, *Tsvetkov* et al. published a landmark study in Science, systematically unraveling and naming its molecular mechanism (Tsvetkov et al., 2022). Cuproptosis, a metal-dependent pathway of cell death, is triggered by excessive copper exposure and subsequent proteotoxic stress. The mechanism involves intracellular copper targeting and binding to lipoylated components in the tricarboxylic acid (TCA) cycle. Aggregation of these copper-bound lipoylated mitochondrial proteins, along with the subsequent reduction in Fe–S (iron–sulfur) clusters, ultimately induces proteotoxic stress and cuproptosis (Chen et al., 2022).

### 3.18 Disulfidptosis

Disulfidptosis, as a new form of PCD triggered by disulfide stress, is characterized by the collapse of cytoskeleton proteins and F-actin due to the intracellular accumulation of disulfides (Zheng et al., 2023). *J Chen* and *B Gan* found that the aberrant accumulation of intracellular disulfides in Solute carrier family 7 member 11 (SLC7A11) - cells under high glucose starvation induces a previously uncharacterized type of cell death, which they term “Disulfidptosis” in 2023 (Liu et al., 2023).

For example, “zinc-induced cell death” currently lacks a formally designated independent cell death mode or academic term within the mainstream classification system (Vitale et al., 2023; Galluzzi et al., 2018) published by the Nomenclature Committee on Cell Death (NCCD). As for now, the term “zinc death” has not been formally proposed in a single paper. Rather, it is a literal translation or simplified expression used by researchers to describe “zinc-induced cell death.” It has not yet been formally recognized as a distinct cell death mode, unlike ferroptosis, necroptosis, and cuproptosis. The expanding landscape of PCD, with its diverse molecular mechanisms and profound implications for disease outcomes, offers a critical theoretical and research framework for understanding how viral infections precisely orchestrate cell death pathways to control viral replication and associated tissue damage.

## 4 Programmed cell death in RSV infection

**Figure 3:**
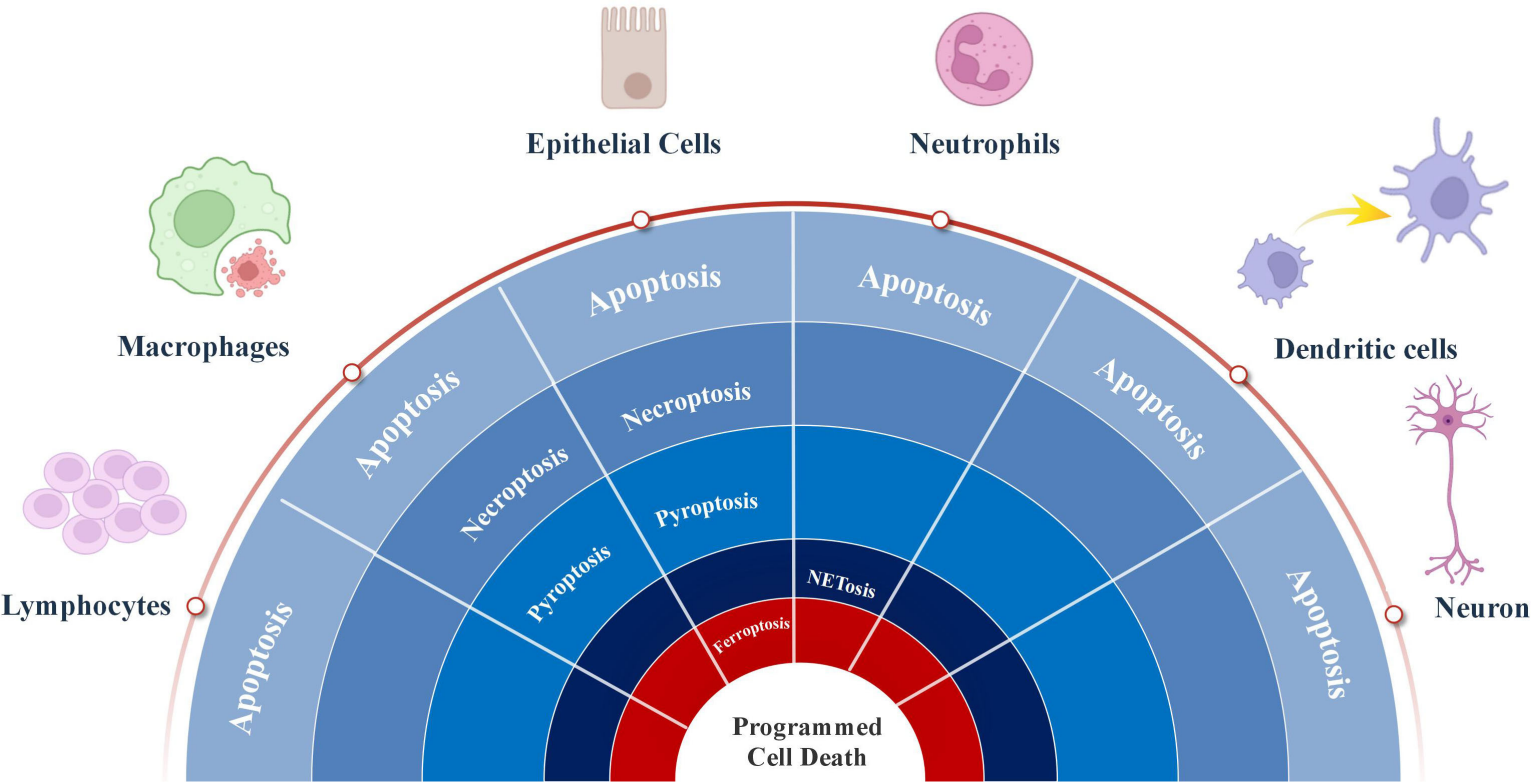
The roles of process of programmed cell death regulated by respiratory syncytial virus in multiple types cells.

In response to viral infection, many cells undergo apoptosis, resulting in a decrease in the release of progeny virus (Rex et al., 2022). Viruses adapt to changes in cell death through various strategies and exploit cell death pathways to facilitate infection and associated tissue damage. The interaction between the virus and cell death is a dynamic interplay between “host clearance” and “viral escape”. Data on RSV and PCD were sourced from key databases, including Elsevier, PubMed, Springer, Google Scholar and Web of Science, covering the period from the inception of the relevant databases to September 2025. Apoptosis, necroptosis, pyroptosis, NETosis and ferroptosis have all been reported in RSV infection (**Figure 3**). This review summarizes current understanding of how RSV regulates PCD and its target cells (**Table 1**).

**Table 1:** Respiratory syncytial virus regulates programmed cell death: forms, target cells, and mechanisms.

No	Programmed cell death types	Cell types	Model	Method	Signal path	Impact (Induce/Inhibit)	Reference
1	Apoptosis	Human ciliated epithelial cells	Human	*In vitro*	/	Induce	(Zhao et al., 2024)
2	Apoptosis	Human laryngeal carcinoma cells HEp-2	Human	*In vitro*	Down-regulation of tumor necrosis factor α-induced protein (TNFAIP3)	Induce	(Martín-Vicente et al., 2020)
3	Apoptosis	Epithelial cells (A549 cells)	Human	*In vitro*	Down-regulation of tumor necrosis factor α-induced protein (TNFAIP3)	Induce	(Martín-Vicente et al., 2020)
4	Apoptosis	Epithelial cells (HEp-2 cells)	Human	*In vitro*	Phosphorylation of tumor suppressor p53	Induce	(Eckardt-Michel et al., 2008)
5	Apoptosis	Epithelial cells (HEp-2 cells)	Human	*In vitro*	/	Induce	(Chi et al., 2022)
6	Apoptosis	Epithelial cells (BEAS-2B cells)	Human	*In vitro*	/	Induce	(Chi et al., 2022)
7	Apoptosis	Epithelial cells (A549 cells)	Human	*In vitro*	/	Induce	(Eckardt-Michel et al., 2008)
8	Apoptosis	Neutrophils	Human	*In vivo*	Up—regulation of CD11b/CD18	Induce	(Wang et al., 2001)
9	Apoptosis	Neutrophils	Human	*In vivo*	/	Induce	(Deng et al., 2020)
10	Apoptosis	Epithelial cells (A549 cells)	Human	*In vitro*	Activation of caspase-12 and NF-kappa B	Induce	(Bitko and Barik, 2001)
11	Apoptosis	Dendritic cells	Human	*In vivo*	/	Induce	(Bartz et al., 2003)
12	Apoptosis	Epithelial cells (A549 cells)	Human	*In vitro*	/	Inhibit	(Bitko et al., 2007)
13	Apoptosis	Neutrophils	Human	*In vitro*	/	Induce	(Robinson et al., 2024)
14	Apoptosis	Dendritic cells	Human	*In vitro*	/	Inhibit	(Le Nouën et al., 2009)
15	Apoptosis	Lymphocytes	Human	*In vitro*	/	Induce	(Qin et al., 2011)
16	Apoptosis	Dendritic cells	Mice	*In vitro*	/	Induce	(Do Nascimento De Freitas et al., 2016)
17	Apoptosis	Macrophages	Mice	*In vitro*	Activation of iNOS	Induce	(Mgbemena et al., 2013)
18	Apoptosis	Macrophages	Human	*In vitro*	/	Inhibit	(Nakamura-Lopez et al., 2015)
19	Apoptosis	Macrophages	Mice	*In vitro*	Up-regulation of Bcl-2	Inhibit	(Nakamura-López et al., 2011)
20	Apoptosis	Neutrophils	Human	*In vivo*	/	Inhibit	(M. Coleman, 2011)
21	Apoptosis	Pulmonary γδ T cells	Mice	*In vivo*	Up-regulation of Factor-related Apoptosis ligand(FASL)	Induce	(Zeng et al., 2014)
22	Apoptosis	CD4+ helper lymphocytes and CD8+ cytotoxic lymphocytes	Human	*In vivo*	/	Induce	(Roe et al., 2004)
23	Apoptosis	CD8+ cytotoxic lymphocytes	Mice	*In vivo*	/	Induce	(Lu et al., 2015)
24	Apoptosis	Epithelial cells (A549 cells)	Human	*In vitro*	Activation of PI3K/ NF-κB pathway	Inhibit	(Thomas et al., 2002)
25	Apoptosis	Neuro-2a cells	Mice	*In vitro*	Up-regulation of TLR4, and C23	Induce	(Hu et al., 2019)
26	Apoptosis	Neuro-2a cells	Mice	*In vitro*	Up-regulation of TLR4, TLR3 and TLR7	Induce	(Yuan et al., 2018)
27	Apoptosis	Neuroblastoma SH-SY5Y cells	Human	*In vitro*	Activation of TLR3 and RIG-I, up-regulation of TNF-α, IL-6, and IL-8	Induce	(Zhang et al., 2021)
28	Necroptosis	Macrophages	Human	*In vitro*	Activation of TLR4, TLR3 and RIPK3/MLKL necroptotic pathway	Induce	(Bedient et al., 2020)
29	Necroptosis	Macrophage	Mice	*In vitro*	Up-regulation of tumor necrosis factor (TNF)	Induce	(Santos et al., 2021)
30	Necroptosis	Airway epithelial cell	Mice	*In vitro*	/	Induce	(Simpson et al., 2020)
31	Necroptosis	Airway epithelial cell	Human	*In vitro*	/	Induce	(Simpson et al., 2022)
32	Necroptosis	Macrophages	Mice	*In vitro*	/	Induce	(Da Silva, 2018)
33	Necroptosis	Macrophages	Mice	*In vitro*	Up-regulation of ATF6	Induce	(Kothwela, 2022)
34	Pyroptosis	Macrophages	Human	*In vitro*	Activation of ASC/NLRP3/caspase-1 pathway	Induce	(Bedient et al., 2020)
35	Pyroptosis	Epithelial cells (BEAS-2B cells)	Human	*In vitro*	/	Induce	(Jiao and Yang, 2025)
36	Pyroptosis	Epithelial cells (16HBE cells)	Human	*In vitro*	/	Induce	(Chen et al., 2022)
37	NETosis	Neutrophils	Human	*In vitro*	Activation of PI3K/AKT, ERK and p38 MAPK	Induce	(Muraro et al., 2018)
38	NETosis	Epithelial cells (A549 cells)	Human	*In vitro*	Activation of PI3K/AKT, ERK and p38 MAPK	Induce	(Muraro et al., 2018)
39	NETosis	Fibroblasts(MRC5 cells)	Human	*In vitro*	Activation of PI3K/AKT, ERK and p38 MAPK	Induce	(Muraro et al., 2018)

### 4.1 Apoptosis

Apoptosis is the most prevalent form of cell death in RSV infection. It was also the first type of PCD to be documented, both in general and specifically within the context of RSV research. Apoptosis is the key mechanism in RSV pathogenesis with its ability to subvert the apoptotic pathway in host cells. RSV infects and alters the apoptotic fate of a wide range of cells, from structural epithelial cells to defensive immune cells like macrophages and T-cells. This targeted manipulation of cell death is a cornerstone of RSV infection and propagation.

#### 4.1.1 Epithelial cells

Epithelial cells are primarily perceived as a physical barrier and mediator of mucociliary clearance. Apart from its physical barrier function, the epithelial cells play a central role in innate and adaptive immune responses (Dorscheid et al., 2025). As early as before 2000, a large number of studies had confirmed the complex connection between RSV infection of respiratory epithelial cells and apoptosis (O’Donnell et al., 1999). Airway epithelial cells are the main target cells of RSV (Glaser et al., 2019). Apoptosis of lung epithelial cells serves as a major pathogenic event in the pathogenesis of RSV-induced lung injury. Furthermore, this contributes to the loss of the alveolar capillary barrier, thereby increasing lung permeability and resulting in lung edema (Van Den Berg et al., 2015).

A549 cells, a tumor cell line with properties of normal airway epithelial cells, are a widely recognized *in vitro* cell model for RSV infection. This model shows (Rajan et al., 2022). Nuclear factor kappa-B (NF-κB) has the ability to directly modulate both pro-survival and apoptotic responses (Sherekar et al., 2023). It is also a downstream component of the phosphatidylinositol 3-kinase (PI3K)/protein kinase B(PKB/AKT)pathway. The prevention of apoptosis in airway epithelial cells may serve to preserve host cell integrity until the virus’s replication phase is completed. RSV inhibits A549 apoptosis by activates NF-κB through a PI3K-dependent pathway (Thomas et al., 2002). However, the necrosis and destruction of ciliated epithelial cells is one of the characteristics of RSV bronchiolitis (Guo-Parke et al., 2013). The apoptosis of RSV-infected ciliated cells promotes virus spread and the ongoing process of apoptotic in extruded ciliated cells provides a time window for these cells to spread RSV (Zhao et al., 2024). Both inhibition and promotion of epithelial cell apoptosis occur, but are differentially phase: inhibition of apoptosis in the early stages and promotion of apoptosis in the later stages. The flow cytometry expression showed that RSV infection did not induce apoptosis in A549 cells at 24 h postinfection (Singh et al., 2007). More experiments are needed to confirm the precise timing of cell apoptosis, and it seems unlikely that *in vitro* studies alone can fully address the need.

As an epithelial cell line, HEp-2 cell line was obtained in 1951 from a patient presenting with a laryngeal carcinoma (Gorphe, 2019). The BEAS-2B cell line is a widely used immortalized but non-tumorigenic human cell line established from normal human bronchial epithelium obtained from a non-cancerous individual by Curtis C. Harris’ group in 1988 (Han et al., 2020). RSV infection leads to an increased inflammatory symptoms and apoptosis in the HEp-2 cells and BEAS-2B cells through mechanisms involving ROS generation (Chi et al., 2022). RSV-induced overproduction of ROS is the central mechanism that activates the epidermal growth factor receptor (EGFR) pathway, and promotes the release of pro-inflammatory cytokines (TNF-α, IL-6, IL-1β, IL-18).

#### 4.1.2 Immune cells

Macrophages play a key role in the early innate immune and inflammatory responses to viral pathogens and act as the main cells that participate in RSV infection. Macrophages are polarized during RSV infection, forming two macrophage phenotypes termed as M1-like and M2-like macrophages, involved in the modulation of inflammatory responses (Churiso et al., 2022). The inducible nitric oxide synthase(iNOS) expression/activity is required for optimal apoptosis during RSV infection, and RSV induces macrophage apoptosis via activating iNOS (Mgbemena et al., 2013). The apoptosis involves caspase-dependent and caspase-independent pathways and occurs via intrinsic and extrinsic pathways. Although viral genome encodes 11 viral proteins, RSV phosphor-protein (P) shares a 52% homology with the caspase-8 death domain. RSV P-protein impairs extrinsic apoptosis pathway in a macrophage-like cell line persistently infected with RSV (Nakamura-Lopez et al., 2015). RSV suppresses macrophage apoptosis through Bcl-2 upregulation and manipulation of the intrinsic pathway, mediated by enhanced mitochondrial respiration and the sequestration of pro-apoptotic proteins to stabilize the mitochondrial membrane (Nakamura-López et al., 2011).

Neutrophils are the predominant airway leukocytes in RSV infections and their products are likely to play an important role in viral infection (Wang et al., 2001). In fact, neutrophil apoptosis has already occurred in the early stages of RSV infection. At 72 hours, post-RSV infection, neutrophil exposure led to an increase in the numbers of apoptotic neutrophils *in vivo* (Deng et al., 2020). The apoptosis of neutrophils in RSV infection showed age-related differences, greater numbers of infant neutrophils became apoptotic when compared with adult (Robinson et al., 2024).

Dendritic cells (DCs) are a type of professional antigen-presenting cell. Immature DCs have strong migratory capacity; mature DCs can effectively activate naive T cells and initiate the body’s adaptive immune response in a timely and appropriate manner. RSV induced increased apoptosis in immature DCs. It is conceivable that early apoptosis of RSV-infected DCs could prevent efficient T-cell activation and could account for suppression of cell-mediated immunity (Bartz et al., 2003). RSV can have a negative influence on DCs, causing their apoptosis, which prevents active presentation of foreign antigens and T cell activation. Apoptosis also occurs in mature DCs, and RSV-infected bone marrow dendritic cells (BMDCs) show increased apoptotic cell death in mature DCs (Do Nascimento De Freitas et al., 2016).

Lymphocytes are cells with immune recognition function. RSV can infect human lymphocytes during the immune response to viral challenge (Roberts and O’Banion, 2025). RSV infection induces significant lymphocyte proliferation and accelerated apoptosis (Qin et al., 2011). CD^4+^ helper lymphocytes and CD^8+^ cytotoxic lymphocytes are key players in the immune response against viral infection. In RSV infection, both CD^4+^ and CD^8+^ lymphocytes are primed to undergo apoptosis (Roe et al., 2004; Lu et al., 2015). γδ T cells develop in the thymus from progenitor T cells originating from bone marrow hematopoietic stem cells (Hu et al., 2023). In an Ovalbumin (OVA)-sensitized murine model, RSV infection influences FasL-mediated apoptosis of pulmonary γδ T cells. This effect partly suppresses the subsequent development of OVA-induced allergic responses (Zeng et al., 2014).

#### 4.1.3 Other cells

Growing evidence links RSV infection to neurological manifestations, such as febrile seizures, encephalopathy, and less frequently, encephalitis or meningoencephalitis (Stravoravdi et al., 2025). Neuro-2a(N2a) cells, a rapidly growing mouse neuroblastoma cell line, were derived from a spontaneous tumor in an albino strain A mouse. RSV prolifically entered and infected N2a neuronal cells, leading to the modulated expression of Toll-like receptor 4 (TLR4) and C23, as well as of TLR3, TLR7, and their downstream inflammatory factors, suggesting a direct induction of RSV-associated encephalopathy in infants by the infection of neuronal cells (Yuan et al., 2018; Hu et al., 2019). The SH-SY5Y human neuroblastoma cell line, like the N2a cell line, allows the study of the nervous system-related diseases. RSV infection of microglia induces SY5Y neuronal injury and apoptosis via the release of inflammatory cytokines (TNF-α, IL-6, and IL-8) and the upregulation of TLR3 and RIG-I expression (Zhang et al., 2021).

### 4.2 Necroptosis

Necroptosis has been implicated as a critical cell death pathway in virus-infected cells (Cotsmire et al., 2021). RSV has been shown to trigger necroptosis in an infected lungs. Necroptosis blockade may represent a therapeutic strategy for a range of lung pathologies with viral etiologies (Gautam et al., 2024).

RSV infection is a complicated process, several cell types are implicated in the disease progression. As the primary barrier against respiratory pathogens, the airway epithelium can be bypassed by viruses via various means, with their most straightforward approach being the direct infection of epithelial cells. RSV infection readily induces necroptosis in RSV-infected airway epithelial cells. This is detrimental to viral clearance and accentuates immunopathology and the ensuing propensity to develop asthma (Harker and Snelgrove, 2020). RSV infection of airway epithelial cells induces phosphorylation of necroptosis proteins prior to the induction of cell death. RSV infection induced an “early” (6 hpi) and “late” (24 hpi) phosphorylation of receptor-interacting protein kinase 3 (RIPK1)/receptor-interacting protein kinase 3 (RIPK3)/mixed-lineage kinase domain-like (MLKL). However, necroptotic cell death was restricted to the late phase (Simpson et al., 2022). Inhibition of necroptosis may be a viable strategy to limit the severity of viral bronchiolitis and break its nexus with asthma (Simpson et al., 2020).

Macrophages may exacerbate the severity of RSV-induced disease, and necroptosis is closely associated with this process. One of the key factors driving exaggerated lung inflammation during RSV infection is the necroptosis of RSV-infected immune cells, particularly macrophages. The RIPK1-dependent necrotic cell death was termed “Necroptosis”. Despite morphological similarities, it is distinct from necrosis, which is an accidental cell death caused by irreversible cellular injury. RIPK3 and MLKL are the key downstream molecules of RIPK1, with MLKL serving as the ultimate executor of necroptosis (Makuch et al., 2024). During RSV infection of macrophages, viral replication promotes tumor necrosis factor (TNF) production and secretion; TNF then binds to TNF receptor 1 (TNFR1), triggering RIPK1/RIPK3 phosphorylation, subsequent MLKL activation, and ultimately leading to macrophage necroptosis (Santos et al., 2021). Previous studies have confirmed that macrophages undergo necroptosis during RSV infection, a process that depends on RIPK3 and MLKL (Da Silva, 2018; Bedient et al., 2020). RIPK3 forms a complex called the necrosome, which then recruits and phosphorylates MLKL. This triggers MLKL oligomerization, enabling the formation of pore-like structures in the plasma membrane that execute necroptosis (Shi et al., 2023). Persistent or aberrant ER stress induces cell injury (e.g., apoptosis, necroptosis), whereas activating transcription factor 6 (ATF6)—acting as a transcription factor—induces ER chaperone proteins (Li et al., 2023). Notably, the ATF6-dependent ER stress pathway plays a positive role in RSV-mediated necroptosis of macrophages, and this effect is exerted via the induction and activation of ATF6 protein in RSV-infected macrophages (Kothwela, 2022).

### 4.3 Pyroptosis

Pyroptosis plays a pivotal role in eradicating intracellular pathogen replication niches and modulating the immune system via the release of danger signals. Pyroptosis is an important innate immune response that is critical in fighting infections (Wei et al., 2022). Viruses precisely activate or inhibit host pyroptotic pathways, thereby evading immune surveillance, promoting dissemination, and establishing persistent infections (Zhang et al., 2025). Previous studies confirm that RSV infection induces severe inflammation and lung tissue damage via pyroptosis. The Nod-like receptor protein containing pyrin 3(NLRP3) inflammasome-induced pyroptosis pathway contributes to enhanced lung immunopathology during RSV infection and promotes the development of RSV-associated asthma (Lin and Porto, 2025). The NLRP3 inflammasome leads to caspase-1 activation and the cleavage of different cellular proteins, including pro-interleukin (IL)-1β and pro-IL-18, as well as gasdermin D (GSDMD). The resulting GSDMD N-terminal fragment oligomerizes in the plasma membrane, forming GSDMD pores, inducing large ruptures in the membrane, thereby leading to pyroptosis (Schachter et al., 2025).

Epithelial cells are structural cells that maintain the stability of lung function and serve as an important battlefield for RSV infection. The human bronchial epithelial cell line, 16HBE14o- (16HBE), is widely used as a model for respiratory epithelial diseases and barrier function (Callaghan et al., 2020). RSV can induce 16HBE cell pyroptosis, thereby promoting viral replication and spread (Chen et al., 2022). BEAS-2B cells are immortalized cells derived from normal human bronchial epithelial cell. Electron microscopy was used to assess morphology changes associated with RSV-induced pyroptosis in BEAS-2B cells, including mitochondrial swelling and nuclear disruption. RSV infection elevates the expression of pyroptosis markers, such as NLRP3, apoptosis-associated speck-like protein(ASC), and cleaved caspase-1 (Jiao and Yang, 2025). Macrophage pyroptosis plays a critical role in virus infected pathophysiology. The caspase-1 dependent pyroptosis pathway, specifically the ASC/NLRP3/caspase-1 axis, serves as a key mechanism promoting lytic cell death in RSV-infected macrophages, while infection-generated ROS positively regulate this lytic cell death (Bedient et al., 2020).

### 4.4 others

NETosis is a special form of PCD with remarkable specificity. Neutrophils are one of the host’s first defensive measures against viral infection. Neutrophils employ three major defensive mechanisms, including phagocytosis, degranulation and NETosis (Sebina and Phipps, 2020). NETs can capture and kill RSV, contributing to host defense. Extensive neutrophil accumulation in the lungs is a distinct feature of severe RSV-LRTD, and these activated neutrophils release NETs (Cortjens et al., 2016). Neutrophils recognize the fusion protein of RSV via TLR4, subsequently releasing NETs (Hong et al., 2022). ROS are essential for NET formation or NETosis, excess ROS produced during neutrophil activation induces NETosis by inducing extensive DNA damage, and the subsequent DNA repair pathway, leading to chromatin decondensation. RSV can induce classic ROS-dependent NETosis in neutrophils (Muraro et al., 2018).

Numerous studies have demonstrated that ferroptosis plays a significant regulatory role in various respiratory infectious diseases (Hong et al., 2025). RSV-infected mice exhibit increased secretion of the pro-inflammatory chemokines CCL5 and CCL3, elevated mitochondrial iron content, and upregulated expression of 12/15-lipoxygenase (12/15-LOX, which catalyzes the deoxygenation of polyunsaturated fatty acids [PUFAs]). All these changes correlate with increased activation of the 12/15-LOX signaling pathway (Salimi et al., 2017). The cellular damage observed following RSV infection is mediated by the activation of ferroptosis, and these findings indicate that RSV acts as a trigger for ferroptosis (Kombe Kombe et al., 2024).

## 5 Discussion

### 5.1 Current status and research gaps in RSV-regulated PCD

PCD is considered a key player in a variety of cellular processes that help to regulate tissue growth, embryogenesis, cell turnover, immune response, and other biological processes (Kari et al., 2022). Although the forms of cell death are becoming increasingly diverse and studied, existing evidence suggests that RSV-induced PCD consists of only 5 forms: apoptosis, necroptosis, pyroptosis, NETosis, and ferroptosis. Given the diversity of cell death forms, it is reasonable to assume that many viral strategies to promote/inhibit cell death remain unexplored. Compared with the related research on other viral infections and PCD, there are still many gaps in the research on RSV and PCD. For example, like RSV, Severe Acute Respiratory Syndrome Coronavirus 2(SARS-CoV-2) coronavirus infection also modulates multiple PCDs, such as necroptosis, apoptosis, pyroptosis (Liang et al., 2024), NETosis (Zhu et al., 2022), ferroptosis (Wang et al., 2022). However, it can also regulate lysosomal cell death (Sun et al., 2022), PANoptosis (Yang et al., 2025) and cuproptosis (Wang et al., 2024). These PCD forms have not been reported in RSV studies. Related research on other viruses regulating host cell death can also provide new research ideas and references for RSV intervention in cell death. As a virus with a long history, the complex relationship between RSV and cell death can also provide reference for emerging viruses.

### 5.2 RSV viral proteins induce and inhibit PCD

Induction or inhibition are two distinct relationships between RSV and PCD, their dynamics remain a unresolved question. Different PCD forms, cell types, and stages of infection will lead to different RSV induction/inhibition interventions. However, the relatively stable structure of RSV seems to provide some theoretical basis for this issue. For example, the inhibition/induction of apoptotic mechanism of RSV seems to be related to viral proteins. The RSV genome encodes 11 viral proteins, including viral membrane glycoproteins (G), fusion proteins (F), and small hydrophobic proteins (SH). The crucial function of the SH protein is the inhibition of apoptosis through the suppression of TNF-alpha signaling, leading to the attenuation of caspase-8 activity (Okura et al., 2024). Furthermore, the RSV P-protein impairs the extrinsic apoptosis pathway in a macrophage-like cell line persistently infected with RSV (Nakamura-Lopez et al., 2015). The nonstructural NS proteins of RSV suppress premature apoptosis of the infected cell by a mechanism that is independent of suppression of interferon pathway by these proteins (Bitko et al., 2007). F protein expression can induce caspase-dependent cell death, suggesting its role as a potent inducer of apoptosis in RSV-infected cells (Faghirabadi et al., 2024). However, the binding forms and dominance of these proteins across different PCDs and different cells types remain largely unknown.

### 5.3 Crosstalk between cell death pathways in RSV infection

Studies over the years have revealed the distinctive molecular mechanisms and functional consequences of several key cell death pathways in RSV infection. Whether for host cells or viruses, PCD is a double-edged sword, playing the roles of both “protector” and “executioner”. Crosstalk is an unavoidable phenomenon in cell death research. Multiple cell death pathways, including apoptosis, necroptosis, pyroptosis, ferroptosis, and autosis, does not function in isolation but rather form a highly interconnected regulatory network. They frequently share the same signaling molecules, upstream triggers, and downstream effectors. RSV infection is a clear example; for example, RSV can induce the classical ROS-dependent NETosis through necroptosis pathways activation (Muraro et al., 2018). The crosstalk mechanisms between different forms of cell death, such as apoptosis and necroptosis (Rojas-Rivera et al., 2024), necroptosis and pyroptosis (Frank and Vince, 2019), and apoptosis and ferroptosis (Sun et al., 2021), have been reported. However, similar studies are rarely reported in RSV infection. The specific molecular interactions and crosstalk relationships among these PCDs in RSV infection are still relatively limited, and a systematic understanding has not yet been formed.

### 5.4 Different cell-specific PCDs in RSV infection

RSV induces distinct PCD patterns across various cell types, reflecting cell-specific functions in host defense and viral pathogenesis. Epithelial cells, the primary targets of RSV, exhibit stage-dependent apoptosis: early infection inhibits apoptosis to support viral replication, whereas late infection promotes apoptosis for viral spread. Macrophages show dual apoptotic regulation during the RSV infected process. NETosis is a program for formation of NETs, and both NETosis and apoptosis can occur in RSV-infected neutrophils. These differences arise from cell-specific functions and RSV’s tailored manipulation of PCD pathways. RSV primarily infects the respiratory epithelium links to neurologic sequelae with proof of previous studies (Pollard et al., 2024). PCD of neuronal cells confirms the mechanism of RSV nerve damage and provides a reference for Virus Infections in the nervous system, but the number of related studies is still insufficient.

## 6 Conclusion

In conclusion, the systematically review on previous studies revealed the diverse effects of RSV infection on PCD in host cells, showing on how RSV induces PCD, leads to tissue damage, inflammation, and severe disease, while suppressing PCD to evade physical and immune clearance. Although the details and mechanisms of RSV-induced PCD in different host cells remain incompletely understood, regulating PCD has been shown to be a crucial weapon in the battle between RSV and host cells. Further research is recommended to discover the detailed relationship between RSV and host cell-associated PCD and identify effective strategies to regulate dysregulated PCD, providing insights for RSV prevention and treatment.
